# Asphyxiated Female and Male Newborn Piglets Have Similar Outcomes With Different Cardiopulmonary Resuscitation Interventions

**DOI:** 10.3389/fped.2020.602228

**Published:** 2020-12-03

**Authors:** Seung Yeon Kim, Gyu-Hong Shim, Megan O'Reilly, Po-Yin Cheung, Tze-Fun Lee, Georg M. Schmölzer

**Affiliations:** ^1^Neonatal Research Unit, Centre for the Studies of Asphyxia and Resuscitation, Royal Alexandra Hospital, Edmonton, AB, Canada; ^2^Department of Pediatrics, Eulji University Hospital, Daejeon, South Korea; ^3^Department of Pediatrics, Inje University Sanggye Paik Hospital, Seoul, South Korea; ^4^Department of Pediatrics, University of Alberta, Edmonton, AB, Canada

**Keywords:** infants, newborn, neonatal resuscitation, chest compressions, sex differences

## Abstract

**Background:** Male newborns have a greater risk of poor cardiovascular and respiratory outcomes compared to females. The mechanisms associated with the “male disadvantage” remains unclear. We have previously shown no difference between male and female newborn piglets during hypoxia, asphyxia, resuscitation, and post-resuscitation recovery. However, it is unknown if there are differences in resuscitation outcomes between males and females during different cardiopulmonary resuscitation techniques.

**Intervention and Measurements:** Secondary analysis of 184 term newborn mixed breed duroc piglets (1–3 days of age, weighing 2.0 (0.2) kg) from seven different studies, which were exposed to 30–50 min of normocapnic hypoxia followed by asphyxia until asystole. This was followed by cardiopulmonary resuscitation. For the analysis, piglets were divided into male and female groups, as well as resuscitation technique groups (sustained inflation, 3:1 compression-to-ventilation ratio, or asynchronous ventilations during chest compressions). Cardiac function, carotid blood flow, and cerebral oxygenation were continuously recorded throughout the experiment.

**Main results:** Regardless of resuscitation technique, there was no significant difference between males and females in the number achieving return of spontaneous circulation (ROSC) [95/123 (77%) vs. 48/61 (79%)], the time to achieve ROSC [112 (80–185) s vs. 110 (77–186) s], and the 4-h survival rate [81/95 (85%) vs. 40/48 (83%)]. Levels of the injury markers interleukin (IL)−1ß, IL-6, IL-8, and tumor necrosis factor-α in frontoparietal cortex tissue homogenates were similar between males and females.

**Conclusions:** Regardless of resuscitation technique, there was no significant effect of sex on resuscitation outcome, survival, and hemodynamic recovery in asphyxiated newborn piglets.

## Introduction

It is widely recognized that male newborns are disadvantaged in terms of many health outcomes, particularly cardiovascular and respiratory outcomes, compared to female newborns (1). Male infants experience more postnatal complications, including lower Apgar scores, a greater need for supplemental oxygen, higher rates of respiratory distress syndrome, more pulmonary interstitial emphysema, higher overall perinatal mortality rates, and worse long-term outcomes (1–5). The exact mechanisms associated with biologic disadvantages in males remain unclear. However, obstetric risk factors such as hypoxia, the influence of sex hormones, alterations in cell death pathways and sensitivity to inflammation and excitotoxins, as well as sex differences in autonomic and endocrine stress responses have been suggested to play a role in this biological disadvantage with males (6, 7).

Perinatal asphyxia is one of the most important causes leading to neonatal neuropathy and mortality as well as long-term neurologic impairments and complications such as cerebral palsy, learning disabilities, and intellectual disability among survivors (8). By understanding factors and conditions associated with perinatal asphyxia, it might be possible to find ways to prevent and reduce complications from perinatal asphyxia. Several studies have indicated increased vulnerabilities to birth asphyxia in male newborns (8–10). This is supported by previous human and animal studies that show males have poorer lung maturation, more cardiac defects, and lower cardiac function than females (7, 11–13). Indeed, if male infants are more vulnerable to perinatal asphyxia, more considerable and careful treatment of male infants with asphyxia would be necessary in the delivery room to prevent these adverse outcomes. Even though the male disadvantage is reported by many studies in newborn humans and animals, we have previously shown no difference between male and female newborn piglets during hypoxia, asphyxia, resuscitation, and post-resuscitation recovery (14). Current resuscitation guidelines recommend performing chest compressions (CC) at a rate of 90/min with 30 ventilations (3:1 C:V) to achieve adequate oxygen delivery (15–17). Apart from current recommendations, several interventions, namely CC with asynchronous ventilations (CCaV) and sustained inflation during chest compression (CC+SI), have also been shown to be effective during cardiopulmonary resuscitation (18, 19). The aim of the study was to investigate whether the sex difference will affect the outcomes with different resuscitation techniques in a neonatal porcine asphyxia model. We hypothesized that, regardless of resuscitation technique, there would be no differences between male and female newborn piglets in terms of resuscitation outcome, survival, and hemodynamic recovery post-resuscitation.

## Methods

**Figure 1:**
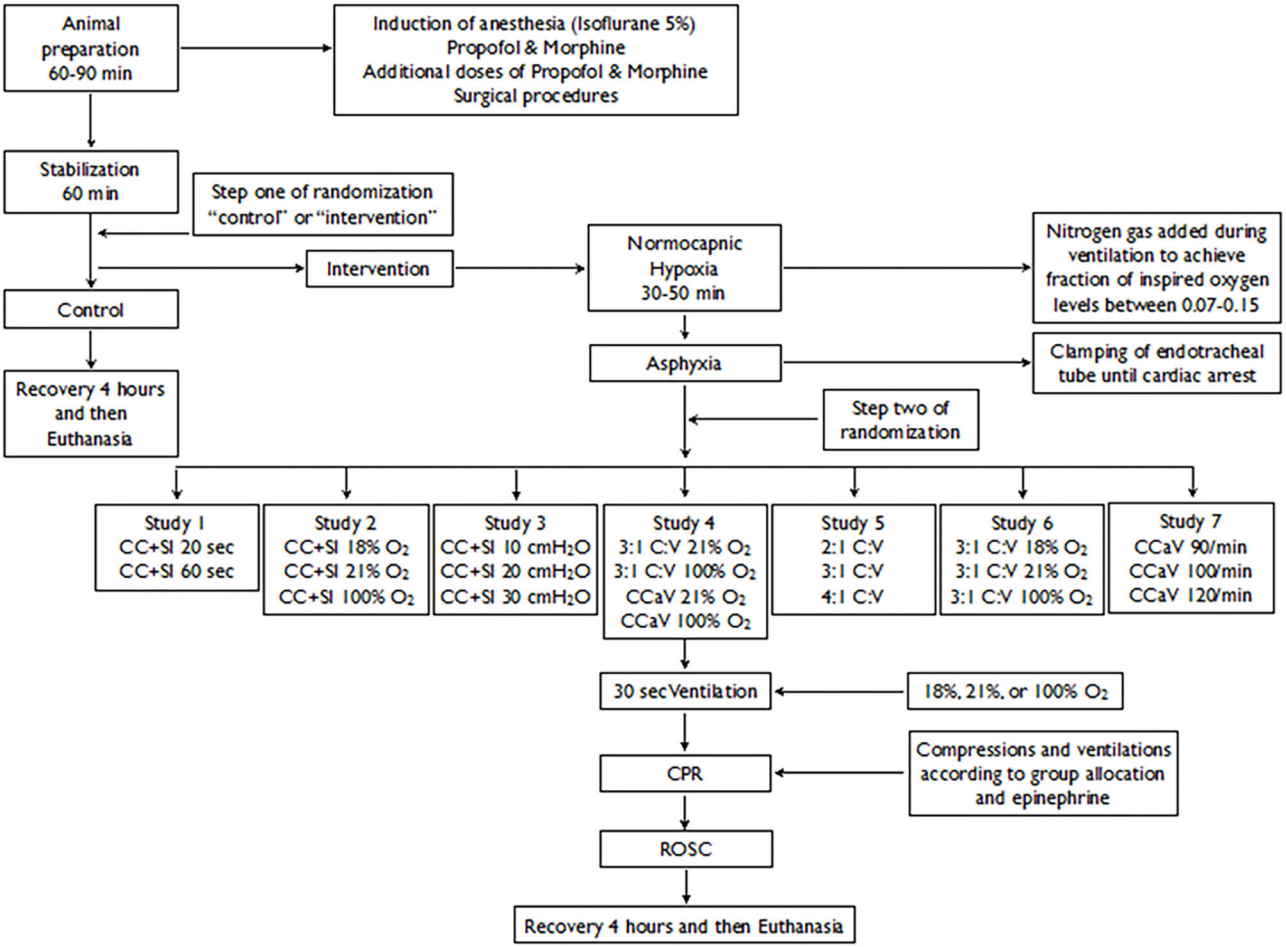
Study Flow Diagram.

Secondary analysis of seven previous publications (20–26). A total of 184 term newborn mixed breed duroc piglets [1–3 days of age, weighing mean (standard deviation) 2.0 (0.2) kg] were obtained on the day of experimentation from the University Swine Research Technology Centre. All experiments were conducted in accordance with the guidelines and approval of the Animal Care and Use Committee (Health Sciences), University of Alberta (AUP00001764, AUP00002151, AUP00002651), presented according to the ARRIVE guidelines (27). The study protocol is presented in Figure 1.

### Animal Preparation

After induction of anesthesia with isoflurane, piglets were intubated via a tracheostomy, and ventilated using pressure-control ventilation (Acutronic Fabian HFO; Hirzel, Switzerland) at a respiratory rate of 16–20 breaths/min and pressure of 20/5 cmH_2_O (20–26). Oxygen saturation was kept within 90–100%, glucose level and hydration maintained with an intravenous infusion of 5% dextrose at 10 mL/kg/h. During the experiment, anesthesia was maintained with intravenous propofol 5–10 mg/kg/hr and morphine 0.1 mg/kg/h. Additional doses of propofol (1–2 mg/kg) and morphine (0.05–0.1 mg/kg) were also given as needed. The piglet's body temperature was maintained at 38.5–39.5°C using an overhead warmer and a heating pad (20–26).

### Hemodynamic Parameters

A 5-French Argyle® (Klein-Baker Medical Inc. San Antonio, TX) double-lumen catheter was inserted via the right femoral vein for administration of fluids and medications. A 5-French Argyle® single-lumen catheter was inserted above the right renal artery via the femoral artery for continuous arterial blood pressure monitoring in addition to arterial blood gas measurements. The right common carotid artery was also exposed and encircled with a real-time ultrasonic flow probe (2 mm; Transonic Systems Inc., Ithaca, NY) to measure cerebral blood flow.

Piglets were placed in supine position and allowed to recover from surgical instrumentation until baseline hemodynamic measures were stable (minimum of 1 h). Ventilator rate was adjusted to keep the partial arterial CO_2_ −35 mmHg as determined by periodic arterial blood gas analysis. Mean systemic arterial pressure, systemic systolic arterial pressure, heart rate, and percutaneous oxygen saturation were continuously measured and recorded throughout the experiment with a Hewlett Packard 78833B monitor (Hewlett Packard Co., Palo Alto, CA).

### Respiratory Parameters

A respiratory function monitor (NM3, Respironics, Philips, Andover, MA) was used to continuously measure tidal volume, airway pressures, gas flow, and end-tidal CO_2_. The combined gas flow and ETCO_2_ sensor was placed between the endotracheal tube and the ventilation device. Tidal volume was calculated by integrating the flow signal (28). End-tidal CO_2_ was measured using non-dispersive infrared absorption technique. The accuracy for gas flow is ± 0.125 L/min, end-tidal CO_2_ ± 2 mmHg (29).

### Cerebral Perfusion

Cerebral oxygenation (crSO_2_) was measured using the Invos^TM^ Cerebral/Somatic Oximeter Monitor (Invos 5100, Somanetics Corp., Troy, MI). The sensor was placed on the forehead of the piglet and secured with wrap and tape. Light shielding was achieved with a slim cap. The Invos^TM^ Cerebral/Somatic Oximeter Monitor calculates crSO_2_, which is expressed as the percentage of oxygenated hemoglobin (oxygenated hemoglobin/total hemoglobin). Values of regional oxygen saturation were stored every second with a sample rate of 0.13 Hz (30).

### Experimental Protocol for Included Studies

*Study 1:* Piglets were randomized to receive chest compressions during sustained inflations (CC+SI) with SI duration of 20 s or 60 s (20). *Study 2:* Piglets were randomized to receive 18%, 21%, or 100% oxygen during CC+SI (26). *Study 3:* Piglets were randomized to receive CC+SI with the SI delivered at to a pressure of 10 cmH_2_O, 20 cmH_2_O, or 30 cmH_2_O (25). *Study 4:* Piglets were randomized to receive 21 or 100% oxygen with asynchronous ventilations during chest compressions (CCaV) or 3:1 compression-to-ventilation ratio (C:V) (22). *Study 5:* Piglets were randomized to receive 2:1, 3:1, or 4:1 C:V (21). *Study 6:* Piglets were randomized to receive 18, 21, or 100% oxygen during 3:1 C:V (24). *Study 7:* Piglets were randomized to receive CCaV delivered at a compression rate of 90, 100, or 120/min (23).

*For all studies:* To reduce the occurrence of selection bias, a two-step randomization process was used. Following surgical instrumentation and the stabilization procedure, a subsequently numbered, sealed brown envelope containing the assignment “sham” or “intervention” was opened (step one). Piglets that were randomized to “intervention” underwent both hypoxia and asphyxia, whereas, the piglets randomized to “sham” were not. Sham-operated groups received the same surgical protocol, stabilization, and equivalent experimental periods without hypoxia and asphyxia. Upon meeting the criteria for resuscitation, a second sequentially numbered, sealed brown envelope, containing the assignment for each study group was opened (step two). The piglets that were randomized to “intervention” were exposed to 30–50 min of normocapnic hypoxia. The piglet was then disconnected from the ventilator and the endotracheal tube was clamped until total cardiac arrest. Fifteen seconds after cardiac arrest, positive pressure ventilation (PPV) was commenced for 30 s with a Neopuff T-Piece (Fisher & Paykel, Auckland, New Zealand). Unless specified by the group allocation, the default settings were a peak inflating pressure of 30 cmH_2_O, a positive end expiratory pressure of 5 cmH_2_O, and a gas flow of 8 L/min. CC were performed using the two-thumb encircling technique by a single operator (GMS) in all piglets. A metronome was used to achieve the targeted CC rate. After 30 s of CC, 100% oxygen was commenced (unless piglets were randomized to receive 18% or 21% oxygen). Epinephrine (0.01–0.02 mg/kg per dose) was administered intravenously 2 min after the start of PPV, and administered every 3 min as needed if no ROSC was observed. Epinephrine was administered to a maximum of four doses. ROSC was defined as an unassisted heart rate >100 bpm, which was accessed by ECG, for 15 s. After ROSC, piglets were allowed to recover for 4 h before being euthanized with an intravenous overdose of sodium pentobarbital (120 mg/kg).

### Data Collection and Analysis

Demographics of study piglets were recorded. Transonic flow probes, heart rate and pressure transducer outputs were digitized and recorded with LabChart® programming software (ADInstruments, Houston, TX). The data are presented as median (interquartile range - IQR). Post-mortem, the brain was removed from the skull and placed in ice-cold 2-methylbutane for 10 min before storing at −80°C. Brain tissue samples were homogenized in a lysis buffer (0.5% Tween-20/PBS containing a protease inhibitor cocktail). Homogenized samples were centrifuged at 3,000 × g for 10 min at 4°C. The supernatants were retained, and protein concentration was quantified using the Bradford method. Concentrations of brain injury markers, the pro-inflammatory cytokines interleukin (IL)-1ß,−6,−8, and tumor necrosis factor (TNF)-α, were determined with commercially available ELISA kits (PLB00B, P6000B, P8000, PTA00, R&D Systems, Minneapolis, USA). Cytokine concentrations were quantified according to protocols provided by the manufacturer and were expressed relative to protein concentration.

### Statistical Analysis

Data are presented as mean ± standard deviation (± SD) for normally distributed continuous variables and median (interquartile range - IQR) when the distribution was skewed. For all respiratory parameters, continuous values during CPR were analyzed. The data was tested for normality and compared using Student's *t-*test for parametric and Mann-Whitney *U*-test for non-parametric comparisons of continuous variables, and χ^2^ for categorical variables. A Kaplan-Meier survival curve was generated to compare outcomes between male and female. *P*-values are 2-sided and *p* < 0.05 was considered statistically significant. Statistical analyses were performed with SigmaPlot (Systat Software Inc, San Jose, USA) and SAS Ver. 9.4 (SAS Institute Inc., Cary, NC, USA).

## Results

**Figure 2:**
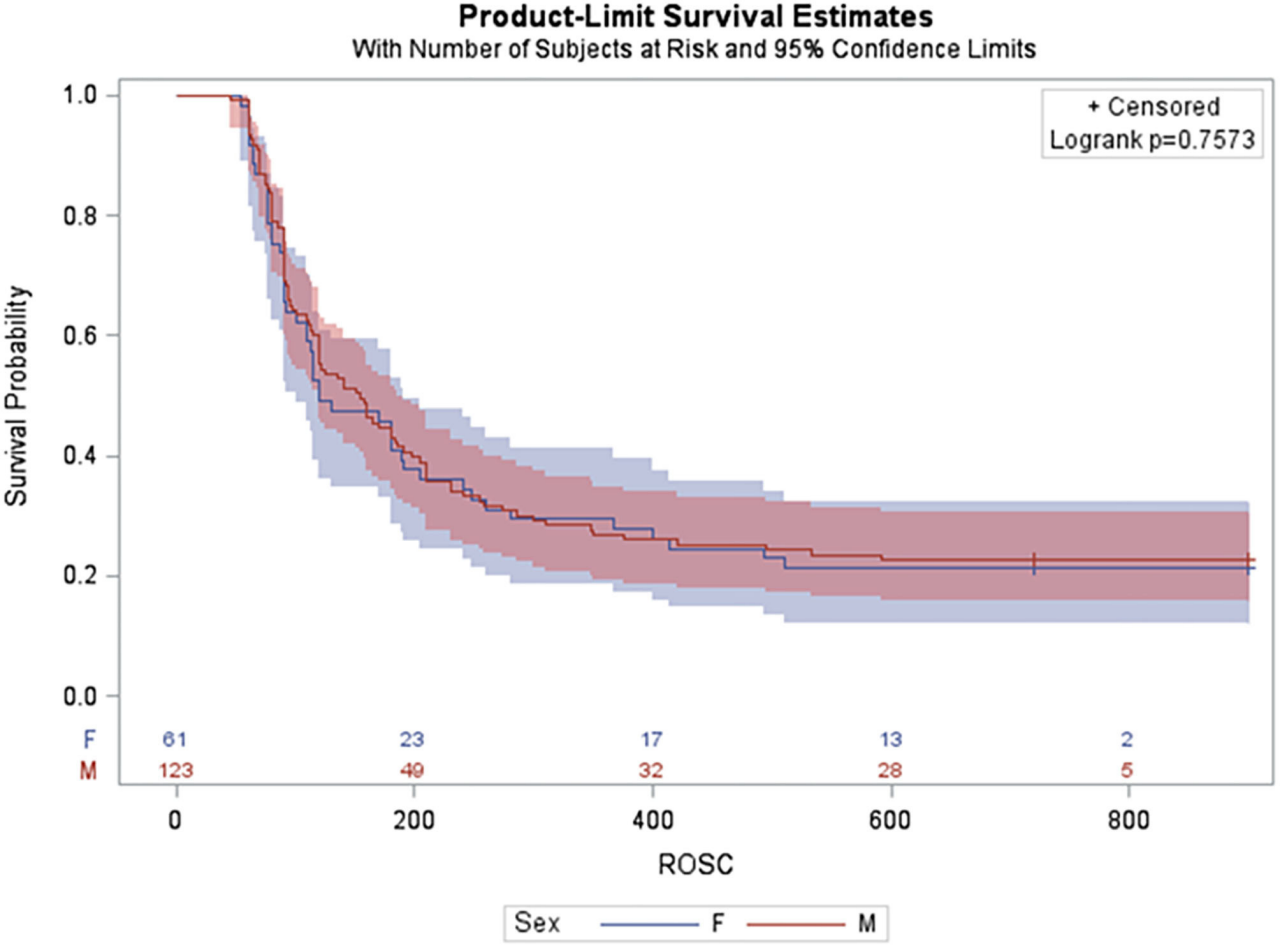
Kaplan-Meier survival curve for male and female.

Newborn mixed breed duroc piglets (*n* = 184) were obtained on the day of the experiment and were subjected to the hypoxia-asphyxia protocol of the specific study. Baseline parameters were similar between male and female piglets (Table 1). Duration of asphyxia (Table 2) and the degree of asphyxiation (as indicated by pH, PaCO_2_, base excess, and lactate; **Table 4**) were similar between male and female piglets. A total of 95/123 (77%) male and 48/61 (79%) female piglets achieved ROSC (Table 2). Median (IQR) time to achieve ROSC in males and females was 112 (80–185) s and 110 (77–186) s, respectively (Table 2). The 4-h survival rate after ROSC was similar between male and female piglets, with 81/95 (85%) male and 40/48 (83%) female piglets surviving (Table 2). A Kaplan-Meier survival curve is presented in Figure 2.

**Table 1:** Baseline characteristics.

	**Overall**		**CC+SI**		**3:1 C:V**		**CCaV**	
	**Male** ** (*n* = 123)**	**Female** ** (*n* = 61)**	**Male** ** (*n* = 61)**	**Female** ** (*n* = 27)**	**Male** ** (*n* = 33)**	**Female** ** (*n* = 23)**	**Male** ** (*n* = 29)**	**Female** ** (*n* = 11)**
Age (days)	2 (1–2)	2 (1–3)	2 (1–2)	2 (1–3)	2 (1–3)	2 (1–3)	2 (2–2)	2 (2–3)
Weight (kg)	2.1 (1.9–2.2)	2 (1.8–2.2)	2.1 (1.9–2.2)	2.0 (1.9–2.1)	2.1 (2.0–2.2)	2.1 (1.8–2.2)	2.1 (1.8–2.2)	2.1 (1.7–2.2)
Heart rate (bpm)	191 (171–222)	197 (171–230)	191 (171–210)	196 (157–223)	194 (168–232)	186 (170–223)	192 (175–230)	222 (184–241)
MAP (mmHg)	63 (57–68)	64 (57–69)	61 (56–66)	59 (54–70)	66 (57–71)	65 (55–68)	66 (58–73)	69 (61–74)
Carotid flow (mL/min)	46 (39–58)	48 (39–58)	46 (36–55)	42 (33–49)	46 (30–57)	40 (27–61)	36 (27–51)	37 (25–56)
Cerebral oxygenation (%)	46 (41–51)	47 (41–51)	49 (43–54)	46 (41–49)	47 (40–61)	51 (45–60)	46 (40–61)	51 (37–67)
pH	7.47 (7.44–7.51)	7.48 (7.42–7.53)	7.49 (7.45–7.55)	7.5 (7.46–7.54)	7.45 (7.41–7.48)	7.46 (7.41–7.53)	7.49 (7.45–7.51)	7.49 (7.42–7.51)
Base excess (mmol/L)	2 (0–4)	2 (0–4)	2 (0–4)	2.5 (0.8–4.3)	1 (-1–4)	1 (-1–3)	2.5 (0.3–4.5)	3 (-1–4)
paCO_2_ (torr)	34 (32–37)	34 (31–37)	34 (31–36)	34 (30–36)	35 (32–38)	35 (31–39)	33 (32–36)	33 (30–37)
PaO_2_ (torr)	88 (79–95)	86 (76–102)	91 (83–99)	94 (76–105)	84 (73–90)	82 (68–102)	94 (78–98)	83 (79–91)
SpO_2_ (%)	97 (97–98)	97 (96–98)	98 (97–98)	98 (97–99)	97 (96–98)	97 (96–98)	98 (96–98)	97 (96–97)
Lactate (mmol/L)	3.9 (3.1–4.5)	3.4 (3.0–4.0)	3.9 (3.1–4.8)	3.3 (2.9–4.0)	3.6 (3.1–4.3)	3.4 (3.1–4.0)	4.2 (3.1–5.1)	3.8 (2.8–4.3)
Arterial hemoglobin (g/L)	76 (70–85)	77 (71–85)	75 (65–81)	76 (69–87)	77 (67–86)	78 (73–87)	75 (73–90)	75 (69–84)

*Data are presented as median (IQR); MAP, Mean arterial blood pressure; CC+SI, chest compression superimposed during sustained inflation; 3:1 C:V, Compression:Ventilation ratio; CCaV, chest compression with asynchronized ventilation*.

**Table 2:** Characteristics of asphyxia, resuscitation, and survival of asphyxiated piglets.

	**Overall**		**CC+SI**		**3:1 C:V**		**CCaV**	
	**Male** ** (*n* = 123)**	**Female** ** (*n* = 61)**	**Male** ** (*n* = 61)**	**Female** ** (*n* = 27)**	**Male** ** (*n* = 33)**	**Female** ** (*n* = 23)**	**Male** ** (*n* = 29)**	**Female** ** (*n* = 11)**
Asphyxia time (s)	265 (106–494)	310 (163–435)	360 (215–555)	315 (250–391)	191 (34–488)	365 (120–546)	55 (32–430)	166 (34–310)
Achieving ROSC^#^	95 (77%)	48 (79%)	46 (75%)	22 (81%)	29 (88%)	18 (78%)	20 (69%)	8 (73%)
ROSC time (sec)	112 (80–185)	110 (77–186)	93 (79–169)	90 (65–143)	124 (90–257)	120 (87–287)	120 (90–178)	100 (83–178)
Epinephrine doses (n)	1 (0–3)	1 (0–3)	1 (0–4)	1 (0–2)	1 (0–2)	1 (0–3)	1 (0–4)	1 (0–4)
Survival 4 h after ROSC^#^	81 (85%)	40 (83%)	44 (96%)	21 (95%)	23 (79%)	12 (67%)	14 (70%)	7 (88%)
Survival time after ROSC (min)	240 (240–240)	240 (240–240)	240 (240–240)	240 (240–240)	240 (240–240)	240 (113–240)	240 (113–240)	240 (240–240)

Data are presented as median (IQR) unless indicated

# *n(%); CC+SI, chest compression superimposed during sustained inflation; 3:1 C:V, Compression:Ventilation ratio; CCaV, chest compression with asynchronized ventilation*.

Regardless of resuscitation technique (SI, 3:1 C:V, CCaV), there was no significant difference between males and females in the percentage achieving ROSC, the time to achieve ROSC, and survival to 4-h post-resuscitation after ROSC (Table 2).

### Changes in Hemodynamic and Blood Gas Parameters

**Figure 3:**
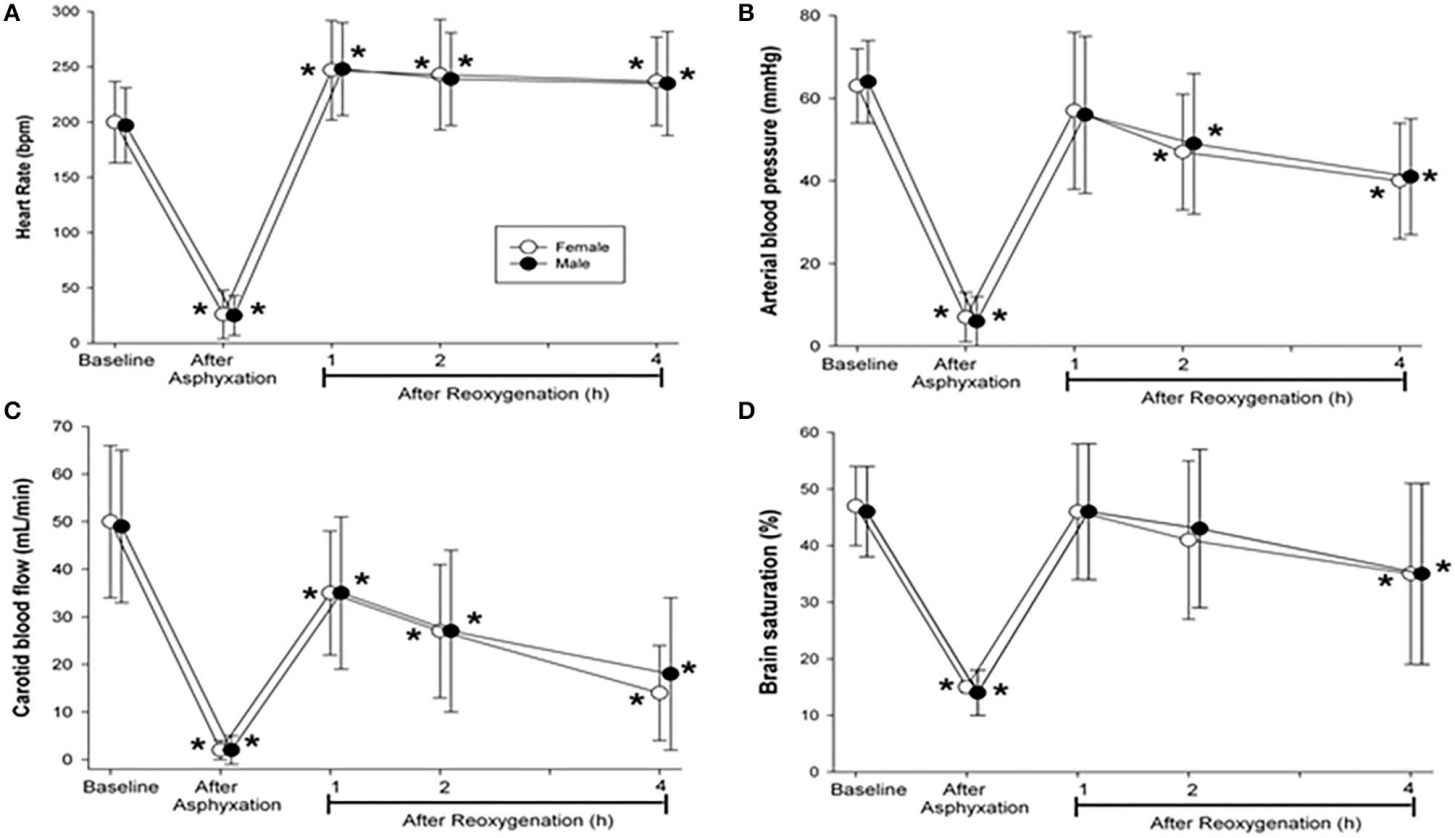
Hemodynamic changes at baseline, after asphyxiation, and 4 h after reoxygenation. Hemodynamic changes in **(A)** heart rate, **(B)** mean arterial blood pressure, **(C)** carotid blood flow, and **(D)** brain oxygen saturation in resuscitated male (closed circle) and female (open circle) piglets. Data are presented as mean (SD). Asterisk (*) indicated a significant difference from baseline values (*p* < 0.05).

Both male and female piglets experienced a significant reduction in heart rate, mean arterial blood pressure, carotid blood flow, and crSO_2_ from baseline values following asphyxia, which was then followed by an increase after resuscitation and ROSC (Figure 3, Table 3). Heart rate remained higher than baseline values for the duration of the 4-h post-resuscitation recovery period (Figure 3A). However, mean arterial blood pressure, carotid blood flow, and crSO_2_ decreased from baseline level throughout the 4-h recovery period (Figures 3B–D). There were no significant differences between male and female piglets in any of the hemodynamic parameters (Figure 3, Table 3).

**Table 3:** Hemodynamic changes at baseline, after asphyxiation, and 4 h after reoxygenation.

	**Overall**		**CC+SI**		**3:1 C:V**		**CCaV**	
	**Male** ** (*n* = 123)**	**Female** ** (*n* = 61)**	**Male** ** (*n* = 61)**	**Female** ** (*n* = 27)**	**Male** ** (*n* = 33)**	**Female** ** (*n* = 23)**	**Male** ** (*n* = 29)**	**Female** ** (*n* = 11)**
**HEART RATE (bpm)**								
Baseline	191 (171–222)	197 (171–230)	191 (171–210)	196 (157–223)	194 (168–232)	186 (170–223)	192 (175–230)	222 (184–241)
After Asphyxiation	24 (16–34)^*^	22 (10–45)^*^	20 (16–28)	19 (11–39)^*^	28 (19–51)^*^	18 (3–51)^*^	27 (0–34)^*^	28 (6–47)^*^
4 h after reoxygenation	247 (213–269)^*^	236 (212–274)^*^	247 (221–268)	262 (192–287)^*^	260 (205–271)^*^	223 (216–245)	230 (191–273)	232 (215–238)
**MAP (mmHg)**								
Baseline	63 (57–68)	64 (57–69)	61 (56–66)	59 (54–70)	66 (57–71)	65 (55–68)	66 (58–73)	69 (61–74)
After Asphyxiation	5 (2–9)^*^	6 (2–10)^*^	7 (2–10)^*^	9 (3–11)^*^	5 (3–8)^*^	6 (0–9)^*^	5 (0–9)^*^	4 (0–11)^*^
4 h after reoxygenation	40 (30–51)^*^	38 (29–50)^*^	39 (34–46)^*^	39 (29–50)^*^	46 (30–57)^*^	40 (27–61)^*^	36 (27–51)^*^	37 (25–56)^*^
**CAROTID BLOOD FLOW (mL/kg)**								
Baseline	46 (39–58)	48 (39–58)	46 (36–55)	42 (33–49)	47 (40–61)	51 (45–60)	46 (40–61)	51 (37–67)
After Asphyxiation	0 (0–5)^*^	0 (0–5)^*^	0 (0–5)^*^	0 (0–5)^*^	5 (0–5)^*^	1 (0–5)^*^	0 (0–5)^*^	0 (0–5)^*^
4 h after reoxygenation	15 (6–27)^*^	14 (5–19)^*^	13 (7–23)^*^	11 (5–19)^*^	24 (7–32)^*^	17 (5–20)^*^	10 (3–21)^*^	14 (5–21)^*^
**BRAIN SATURATION (%)**								
Baseline	46 (41–51)	47 (41–51)	49 (43–54)	46 (41–49)	48 (43–51)	49 (42–54)	42 (36–49)	48 (37–52)
After Asphyxiation	15 (15–15)^*^	15 (15–15)^*^	15 (15–15)^*^	15 (15–15)^*^	15 (15–15)^*^	15 (15–15)^*^	15 (15–15)^*^	15 (15–15)^*^
4 h after reoxygenation	36 (19–50)^*^	34 (16–49)^*^	34 (19–46)^*^	32 (19–44)^*^	38 (22–55)	42 (20–55)	25 (15–43)^*^	33 (15–50)^*^

*Data are presented as median (IQR); MAP, Mean arterial blood pressure*.

* *Significantly different from its own baseline, p < 0.05; CC+SI, chest compression superimposed during sustained inflation; 3:1 C:V, Compression:Ventilation ratio; CCaV, chest compression with asynchronized ventilation*.

Table 4 shows the blood gas parameters (pH, PaCO_2_, base excess, and lactate) at baseline, after asphyxiation, and at 4-h post-resuscitation between male and female piglets. Following asphyxia, pH and base excess values decreased significantly, and PaCO_2_ and lactate values increased significantly compared to baseline values in both male and female piglets (Table 4). Although blood gas parameters recovered gradually over the 4-h post-resuscitation recovery period, by the end of the experiment, pH and base excess remained lower than baseline, and lactate remained higher than baseline (Table 4). PaCO_2_ values returned to baseline by the end of the 4-h post-resuscitation recovery period. At the end of the 4-h recovery period, male piglets resuscitated with SI had a significantly higher lactate value than female piglets resuscitated with SI. However, all other blood gas parameters at all time points were similar between males and females. In summary, there were no differences between male and female piglets resuscitated with either CC+SI, 3:1 C:V, or CCaV in terms of hemodynamic recovery (Table 3) and blood gas changes (Table 4).

**Table 4:** Changes in blood gas parameters at baseline, after asphyxiation, and 4 h after reoxygenation.

	**Overall**		**CC+SI**		**3:1 C:V**		**CCaV**	
	**Male** ** (*n* = 123)**	**Female** ** (*n* = 61)**	**Male** ** (*n* = 61)**	**Female** ** (*n* = 27)**	**Male** ** (*n* = 33)**	**Female** ** (*n* = 23)**	**Male** ** (*n* = 29)**	**Female** ** (*n* = 11)**
**pH**								
Baseline	7.47 (7.44–7.51)	7.48 (7.42–7.53)	7.49 (7.45–7.55)	7.50 (7.46–7.54)	7.45 (7.41–7.48)	7.46 (7.41–7.53)	7.49 (7.45–7.51)	7.49 (7.42–7.51)
After Asphyxiation	6.63 (6.50–6.71)^*^	6.61 (6.50–6.71)^*^	6.59 (6.50–6.68)^*^	6.54 (6.50–6.68)^*^	6.65 (6.51–6.71)^*^	6.64 (6.52–6.71)^*^	6.63 (6.51–6.76)^*^	6.66 (6.55–6.76)^*^
4 h after reoxygenation	7.29 (7.17–7.39)^*^	7.29 (7.24–7.40)^*^	7.24 (7.13–7.42)^*^	7.30 (7.25–7.40)^*^	7.33 (7.17–7.39)^*^	7.33 (7.19–7.39)^*^	7.29 (7.15–7.43)^*^	7.25 (7.15–7.49)^*^
**PaCO**_**2**_ **(TORR)**								
Baseline	34 (32–37)	34 (31–37)	34 (31–36)	34 (30–36)	35 (32–38)	35 (31–39)	33 (32–36)	33 (30–37)
After Asphyxiation	97 (80–115)^*^	100 (84–116)^*^	102 (89–121)^*^	106 (91–118)^*^	96 (72–111)^*^	100 (81–124)^*^	86 (68–107)^*^	90 (65–101)^*^
4 h after reoxygenation	38 (32–44)	38 (33–44)	33 (29–42)	37 (35–41)	41 (24–45)	42 (33–45)	41 (33–48)	33 (30–45)
**BASE EXCESS (mmol/L)**								
Baseline	2 (0–4)	2 (0–4)	2 (0–4)	2.5 (0.8–4.3)	1 (−1~4)	1 (−1~3)	2.5 (0.3~4.5)	3 (−1~4)
After Asphyxiation	−27 (−30~-25)^*^	−29 (−30~-25)^*^	−29 (−30~-25)^*^	−30 (−30~-26)^*^	−26 (−29~-24)^*^	−27 (−30~-25)^*^	−27 (−30~-25)^*^	−28 (−30~-26)^*^
4 h after reoxygenation	−7 (−15~-3)^*^	−5 (−10~-3)^*^	−11 (−17~-3)^*^	−7 (−9~-4)^*^	−5 (−12~-3)^*^	−4 (−12~-2)^*^	−6 (−15~0.5)^*^	−5 (−16~0.5)^*^
**LACTATE (mmol/L)**								
Baseline	3.9 (3.1–4.5)	3.4 (3.0–4.0)	3.9 (3.1–4.8)	3.3 (2.9–4.0)	3.6 (3.1–4.3)	3.4 (3.1–4.0)	4.2 (3.1–5.1)	3.8 (2.8–4.3)
After Asphyxiation	15.6 (14.1–17.1)^*^	15.5 (13.8–17.8)^*^	15.7 (14.2–17.1)^*^	15.3 (13.1–17.2)^*^	15.4 (13.9–17.4)^*^	15.3 (13.8–17.8)^*^	16.0 (14.4–17.0)^*^	15.1 (13.4–18.9)^*^
4 h after reoxygenation	5.8 (3.1–10.3)^*^	4.1 (3.3–6.1)^*^	7.1 (3.5–12.1)^*^	4.0 (3.2–4.9)^#^	4.2 (3.0–8.6)^*^	3.7 (3.3–8.9)^*^	5.6 (2.8–11.1)^*^	5.5 (4.2–9.6)

Data are presented as median (IQR);

* *Significantly different from its own baseline, p < 0.05*;

# *Significantly different from male group at the same time point, p < 0.05; CC+SI, chest compression superimposed during sustained inflation; 3:1 C:V, Compression:Ventilation ratio; CCaV, chest compression with asynchronized ventilation*.

### Injury Markers

There were no differences between males and females resuscitated with either CC+SI, 3:1 C:V, or CCaV in the levels of injury markers IL-1ß, IL-6, IL-8, and TNF-α in frontoparietal cortex tissue homogenates (Table 5). In the sham group, males and females also had similar levels of injury markers in their frontoparietal cortex tissue homogenates (Table 5). However, levels of IL-8 were significantly increased in both male and female piglets that were resuscitated compared to those in the sham group (Table 5). Furthermore, IL-6 was significantly increased in male piglets that were resuscitated compared to those in the sham group (Table 5).

**Table 5:** Injury markers in frontoparietal cortex.

	**Sham**		**with CPR**	
	**Male** ** (*n* = 16)**	**Female** ** (*n* = 7)**	**Male** ** (*n* = 54)**	**Female** ** (*n* = 28)**
IL-1ß (pg/mg protein)	14 (9–22)	17 (7–34)	16 (7–30)	20 (16–29)
IL-6 (pg/mg protein)	2 (0–12)	5 (1–31)	20 (6–61)^*^	11 (4–25)
IL-8 (pg/mg protein)	21 (7–47)	33 (22–118)	79 (35–158)^*^	75 (29–152)^*^
TNF-α (pg/mg protein)	0 (0–0)	0 (0–5)	0 (0–3)	1 (0–11)

*Data are presented as median (IQR)*;

* *Significantly different from sham group, p < 0.05; IL, interleukin; TNF, tumor necrosis factor; CPR, Cardiopulmonary Reanimation*.

### Respiratory Parameters

There was no differences in the respiratory parameters during chest compression (Table 6).

**Table 6:** Respiratory parameters during chest compression.

	**Female**			**Male**		
	**3:1 C:V** ** (*n* = 23)**	**CC+SI** ** (*n* = 27)**	**CCaV** ** (*n* = 11)**	**3:1 C:V** ** (*n* = 33)**	**CC+SI** ** (*n* = 61)**	**CCaV** ** (*n* = 29)**
Tidal volume (mL/kg)	20.8 (2.9)	16.2 (2.8)	13 ()2.8	20.4 (1.8)	13.7 (2.1)	12.8 (1)
Peak inflation pressure (cm H_2_O)	26 (3)	33 (1)	30 (2)	28 (6)	31 (3)	31 (2)
Positive End Expiratory Pressure	5 (0.8)	21 (10)	5 (0.8)	5.3 (0.5)	25 (5.5)	5 (0.9)
(cm H_2_O)						
Exhaled CO_2_ (mm Hg)	18 (6)	18 (4)	26 (8)	13 (12)	19 (8)	32 (15)

*Data are presented as mean (SD); CC+SI, chest compression superimposed during sustained inflation; 3:1 C:V, Compression:Ventilation ratio; CCaV, chest compression with asynchronized ventilation*.

## Discussion

Previously we reported that the percentage achieving ROSC, the time to ROSC, survival to 4-h post-resuscitation after ROSC, as well as hemodynamic measurements were similar between male and female asphyxiated piglets (14). In light of the fact that several new interventions have been introduced for neonatal resuscitation, we evaluated whether the sex differences would have an effect on the outcomes of three types of resuscitation techniques: CC+SI, 3: 1 C:V, and CCaV. Our findings can be summarized as follows: (i) time to ROSC, proportion achieving ROSC, and 4-h survival rates were similar between sexes; (ii) no differences in epinephrine use were observed between sexes; (iii) no differences in any hemodynamic measurements were observed between sexes; (iv) no differences between males and females were observed in the results regardless of the resuscitation technique used; and (v) no differences in brain injury markers and respiratory parameters were observed between sexes.

Female infants are born with improved organ maturation, which translates into improved lung development (31), surfactant phospholipid composition, and surfactant function (7, 13, 32). Providing adequate ventilation to achieve reoxgenation is a cornerstone of neonatal resuscitation, and various resuscitation techniques have been developed in the past decade to improve the functional residual capacity of the lung. A manikin study reported significantly higher minute ventilation, but similar tidal volume, during CCaV as compared 3:1 C:V (33). However, similar tidal volume and minute ventilation were observed in a piglet model of neonatal asphyxia comparing CCaV to 3:1 C:V (34). In contrast to a significant loss of expiratory tidal volume compared to inspiratory tidal volume over each 3:1 C:V cycle, no tidal volume loss was observed in CC+SI. Instead, continuous lung recruitment and establishment of functional residual capacity were observed (35). This improvement in tidal volume delivery has been suggested to result in better alveolar oxygen delivery and lung aeration. Schmölzer et al. achieved passive ventilation with CC, by superimposing uninterrupted CC with SI, which significantly improved hemodynamics, minute ventilation, ROSC and survival compared with 3:1 C:V (36). Similarly in the randomized controlled trial in the delivery room, a significantly shorter time to ROSC in the CC+SI group was observed with higher minute ventilation as compared with the 3:1 C:V group (37). That is, by increasing the intrathoracic pressure and improving minute ventilation with CC+SI, carotid blood flow, mean arterial pressure, % change in ejection fraction, cardiac output, alveolar oxygen delivery and lung aeration may be improved, resulting in faster ROSC (20, 25, 26, 36, 37). Despite differences in various respiratory parameters between the three intervention groups in the current study (CC+SI, 3:1 C:V, CCaV), there were no differences between males and females in ROSC characteristics and survival time. One of the possibilities for the lack of male disadvantage in the current study might be that it was masked by the effect of ventilation, anesthesia, and preparedness for appropriate resuscitation.

Recent experimental studies in rodent models have evaluated neurodevelopmental outcomes after asphyxia, reporting that male rats develop behavioral/neurocognitive deficits more often, compared to female rats (38, 39). In addition, a recent meta-analysis showed that male infants have greater long-term IQ impairment than females with a similar degree of hypoxic-ischemic encephalopathy (40). Hill et al. suggested that sex discrepancy in neonatal hypoxic-ischemic injury may be modulated by the presence of sex specific hormones, sex differences in the preferred mechanisms of apoptosis, and/or the protective effect of X-linked inhibitor of apoptosis on the caspase-dependent apoptotic pathway (41). Although, in the current study, an increase in brain cortex injury markers IL-6 and IL-8 were observed at 4-h post-resuscitation compared to the sham group, no sex differences were observed. We are unable to comment on the neurological outcomes beyond the 4-h window, as all animals were euthanized at that time. Further research is needed to identify sex differences in neurological outcomes after asphyxia.

### Limitations

In addition to the large number of piglets (*n* = 184), our use of a piglet model of asphyxia is a great strength of this translational study, as this model closely simulates delivery room events, with the gradual onset of severe asphyxia leading to bradycardia. However, several limitations should be considered: our model of neonatal asphyxia uses piglets that have already undergone the fetal-to-neonatal transition, and were sedated/anesthetized. Furthermore, our model requires intubation with a tightly sealed endotracheal tube to prevent any endotracheal tube leak; however, this is not the case in delivery rooms where mask ventilation is frequently used, which means there may be more airway leaks and obstructions in real life. Nevertheless, our findings are still clinically relevant as the distribution of cardiac output in the infant immediately at birth and after the transition during asphyxia episodes are qualitatively similar (42). Our resuscitation model is slightly different from the currently recommended resuscitation guidelines, as we administered epinephrine 90 s after CCs were initiated; this may have influenced our results. Nevertheless, there was no significant difference in the number of epinephrine doses administered between groups. The difference in the number of included male and female animals was dependent on availability, which could have influenced our results.

## Conclusion

Overall, there was no significant effect of sex on resuscitation outcome, survival, and hemodynamic recovery in asphyxiated newborn piglets. The similarity in outcomes between male and female newborn piglets was also observed across different resuscitation techniques. Epidemiological studies in the newborn human population are necessary to assure these observations.

## Data Availability Statement

The original contributions presented in the study are included in the article/supplementary materials, further inquiries can be directed to the corresponding author/s.

## Ethics Statement

The animal study was reviewed and approved by all experiments were conducted in accordance with the guidelines and approval of the Animal Care and Use Committee (Health Sciences), University of Alberta (AUP00001764, AUP00002151, AUP00002651).

## Author Contributions

GS, P-YC, MO'R, T-FL, SK, and G-HS: conception, design, collection, assembly of data, analysis and interpretation of the data, drafting of the article, critical revision of the article for important intellectual content, and final approval of the article. All authors contributed to the article and approved the submitted version.

## Conflict of Interest

The authors declare that the research was conducted in the absence of any commercial or financial relationships that could be construed as a potential conflict of interest.

## Abbreviations

CC: Chest compression
CPR: Cardiopulmonary resuscitation
ROSC: Return of spontaneous circulation
MAP: Mean arterial blood pressure.
